# Exploring the role of human-following robots in supporting the mobility and wellbeing of older people

**DOI:** 10.1038/s41598-023-33837-1

**Published:** 2023-04-20

**Authors:** Shuo Li, Kirsty Milligan, Phil Blythe, Yanghanzi Zhang, Simon Edwards, Nic Palmarini, Lynne Corner, Yanjie Ji, Fan Zhang, Anil Namdeo

**Affiliations:** 1 School of Engineering, Newcastle University, Cassie Building, Claremont Road, Newcastle Upon Tyne, NE1 7RU UK; 2 UK’s National Innovation Centre for Ageing, The Catalyst, 3 Science Square, Newcastle Upon Tyne, NE4 5TG UK; 3 Jiangsu Key Laboratory of Urban ITS, Jiangsu Province Collaborative Innovation Centre of Modern Urban Traffic Technologies, School of Transportation, Southeast University, Southeast University Road 2, Nanjing, 211189 China; 4 Department of Geography and Environmental Sciences, Northumbria University, Ellison Place, Newcastle Upon Tyne, NE1 8ST UK

**Keywords:** Civil engineering, Quality of life

## Abstract

With the ever-pressing challenges of societal ageing, robotic technologies for older people are increasingly portrayed as a solution for better independent living for longer. However, the application of human-following robots for elderly citizens has not yet been considered, and any prospective benefits offered by the technology for active ageing have previously been overlooked. This qualitative research aimed to explore older people’s needs and requirements towards the human-following robot through the reflexive thematic analysis of semi-structured interview data from 17 independent older adults, supported by a video-based demonstration of the robot. The results indicate that older people believed that human-following robot has the potential to provide social benefits to an independent older adult by encouraging walking trips and prompting social interaction with others in the community. Practical limitations and cost of the robot are barriers to adoption at present. The findings indicate that there is potential for human-following robots to support active ageing, through increasing opportunities for the social participation of an older adult, but further development of the robot is needed for this potential to be realised.

## Introduction

The world is currently facing the challenge of an increasing ageing population, with the global population of individuals aged 65 or older expected to surpass 1.5 billion by 2050, twice the reported number in 2020^1^. In the UK, it is projected that 24% of the population will be aged 65 or above by 2043, up from 19% at present^2^. The ageing of the population has diverse societal implications, ranging from the provision of pensions and healthcare to the overall accessibility of consumer goods and services^3^. Also, the significant shifts in the ageing population’s composition could pose new challenges for transportation sector, particularly as the travel patterns and requirements of older people are potentially to become increasingly complicated^4^. Mobility and social interaction are essential components of healthy and active aging, which are strongly linked to improved health, wellbeing, and overall quality of life^3,5–10^. It has been generally acknowledged that mobility is strongly associated with older people’s engagement of activities, community involvement, life satisfaction as well as physical and mental health^4,11^. Additionally, it is recognised that physical activity has significant impact on older people’s physical and cognitive health^12^. However, age-related health problems, sensory, cognitive, and physical impairments significantly affect the mobility and physical activities of older individuals^6,13,14^. The ageing process is also strongly associated with social isolation and loneliness, which can adversely affect older people's behavioural, psychological, and physiological wellbeing^15^. Although loneliness can affect individuals of any age, older people are particularly susceptible to experiencing social isolation^16^. To promote healthy aging and support older adults in living safe, comfortable, and independent lives, there is an urgent need to develop innovative technologies that can help them maintain their health and wellbeing, foster social connections with society, communities, family, and friends^6,7,17^.

The rapid development of robotic technology has the potential to provide direct support for older individuals in terms of physical activity, mobility, and social interaction, thereby enabling them to maintain their independence and overall wellbeing^18^. Previous research has studied a wide range of robots that have been created to assist older adults in various ways. These robots can be categorised according to the type of support and assistance they provide to older people.

Previous research has studied robots that are specifically designed to provide physical support to older people and assist them in the daily tasks of independent living. Johnson et al.^19^ explored older people’s requirements towards the mobile service robot. According to their findings, older people required the robot to have features that would enhance their perception of independence and freedom, while also facilitate their social interaction and perception in recreational activities. In addition, by incorporating an innovative robotic arm into the mobile service robot and deploying it at a care facility for older people, Mucchiani et al.^20^ investigated how older people interacted with the robot. The study found that based on the feedback gathered after the interaction, the robot was highly accepted as a useful assistant among older people. Furthermore, a study by Mucchiani et al.^21^ evaluated older people’s interaction with an autonomous mobile robot in everyday situation, using use cases such as water delivery, walking encouragement and pain assessment. Their research revealed that older people regarded the robot as useful, and their level of acceptance grew with increased interaction. Previous studies have also explored the possibility of a robot serving as a walking partner for older people. A study by Karunarathne et al.^22^ investigated whether older people would accept a humanoid robot, the wheeled Roobvie-R3, as a walking partner. The experiment involved 20 older adults aged between 60 and 73 years. The aim of their research was to explore whether the robot would make older people’s walking to be easier and more enjoyable, as well as whether they had a greater intention to walk with it. The results revealed that older people were significantly more inclined to walk with the robot than without it, although there was no notable effect on ease or enjoyment. Notably, some participants likened the robot to a pet, a child/grandchild, or a friend. Additionally, 40% of participants believed that the robot needed additional daily-life functions, which could be addressed through a human-following robot equipped with cargo-carrying capabilities. Their research^22^ has been validated by a later study by Nomura et al.^23^, which also utilised the Roobvie-R3 and found that the robot enhances participants' motivation to walk with it. In contrast to the earlier study, they found that participants derived greater enjoyment from walking with the robot. This appears to contradict the prior findings, but the difference can be explained by the fact that this study examined the robot's use among cognitively impaired adults in a care facility, which was not the case in the earlier literature.

Robot can also play a significant role in supporting the mental and psychological wellbeing of older people by providing companionship, reducing loneliness and social isolation, and facilitating communication between older people and their family and friends. Companion robots resemble humans or animal like have shown potential in improving older people’s mental and psychological health. For example, Chen et al.^24^ deployed a humanoid companion robot (Kabochan) in several long-term care facilities. They have found the humanoid companion robots are potentially effective in reducing short-term neuropsychiatric symptoms and improving the quality of life of older people with dementia while also reducing the burden on their caregivers. Their study emphasized that it is crucial to acknowledge that the use of robots should be customized to meet the needs and requirements of different end-users, and this process necessitates ongoing observation. The reason being, the efficacy of robot-assisted applications can vary from one individual to another, and not everyone may react positively^24^. Also, Robinson et al.^25^ evaluated a companion robot (Paro) by exploring older people’s interaction with it in a residential care facility. They found interacting with the robot significantly reduced the systolic and diastolic blood pressure among older people and concluded that animal-shaped companion robots had positive effects on the health of older people, similar to live animals.

Furthermore, previous research has explored the robots designed for health and safety monitoring. Such robots can support older individuals by observing and analysing their health and safety conditions and responding accordingly. Tseng et al.^26^ designed a robotic system that can track the health conditions of older people according to clinical and medical knowledge and expertise. The robot is integrated with an user-friendly interface for older people and is capable to transmit important or emergency notifications to caregivers automatically. Older people in the study provided highly positive feedback for the system. In addition, Broadbent et al.^27^ explored the design requirements of health-care robots with the key stakeholders, including residents, managers and caregivers in a retirement community. Their research revealed that robot functions such as fall detections, signalling for help, lifting, and monitoring location were perceived as most useful by key stakeholders. Moreover, Sumiya et al.^28^ designed a mobile robot for providing real-time detection of falls among older people and facilitating immediate communication with their family and helpers. The robot was found to be able to significantly increase the fall detection rate compared to traditional fall detection methods using fixed sensors.

## Statement of the problem and research gaps

The review of existing research about robot and olde people suggest that the rapid development and swift progress of robotic technologies potentially enhance their quality of life and benefit their health and wellbeing. However there remain significant areas that necessitate further research.

To begin with, previous research on robots and older people has mainly focused on examining human–robot interaction in indoor environments, such as households or healthcare facilities. While it is certainly important to ensure older people live independently and maintain their health within their own homes or healthcare facilities, outdoor activities such as walking also play a critical role in promoting the physical and psychological wellbeing of older people^11,29^. Walking has been recognised as an active mode of mobility, involving low-intensity physical activity for residents and it offers significant economic, safety, environmental, social and health benefits as a means of transportation^11^. One of the prevailing trends in contemporary robotics is the collaboration between humans and robots, where robots serve as collaborative assistants and partners in shared tasks, one important use case is the human-following scenario where a human and a robot collaborate on a common task or a daily activity that requires the robot following the movements of the human^30^. However, it remains unclear what the needs and requirements of older people are when it comes to utilizing human-following robots for outdoor walking activities. Though there has been extensive research on the use of robots for older adults, there is a trend of focusing on older people with cognitive or physical impairments, and the existing literature often fails to acknowledge the older people as a non-homogeneous group^6,31,32^. While many studies have demonstrated the effectiveness of robots in medical and care-home settings, there is still a lack of research on the use of human following robots to support older individuals living independently in the community. Secondly, although some previous research discovered that robot innovations, such as companion robots, have the potential to enhance social inclusion and reduce loneliness among older people, there is an important concern with such companion robots. These robots mainly offer companionship through human–robot interactions, which could potentially diminish and displace human interaction and relationships^33^. It is still unclear about whether human-following robots can be utilized and designed to enhance the existing social relationships of older people and encourage new social connections and interactions with others within their communities when walking in outdoor environment. Moreover, to date, there are limited research adopting a human-centred design and incorporating the older people in the design and evaluation process of outdoor human-following robots. Knowledge regarding how an age-friendly human-following robot could be designed to meet older persons’ requirements and integrate into their daily lives to support healthier ageing is significantly under-researched.

It is therefore imperative to address the above knowledge gaps which will ensure that development, production, and business strategies for human following robots are optimised for the benefit for both individuals and society. The potential consequences for neglecting to do so may result in the robots being unusable and inaccessible for older people, thereby hindering their expected benefits in helping older people live independently and healthily for longer periods of time.

### Purpose of this study

To fill the research gaps identified above, this paper aims to explore older people’s attitudes, needs and requirements towards the human-following robot in order to provide new knowledge for facilitating the design of age-friendly human-following robots.

## Method

### Apparatus

**Figure 1:**
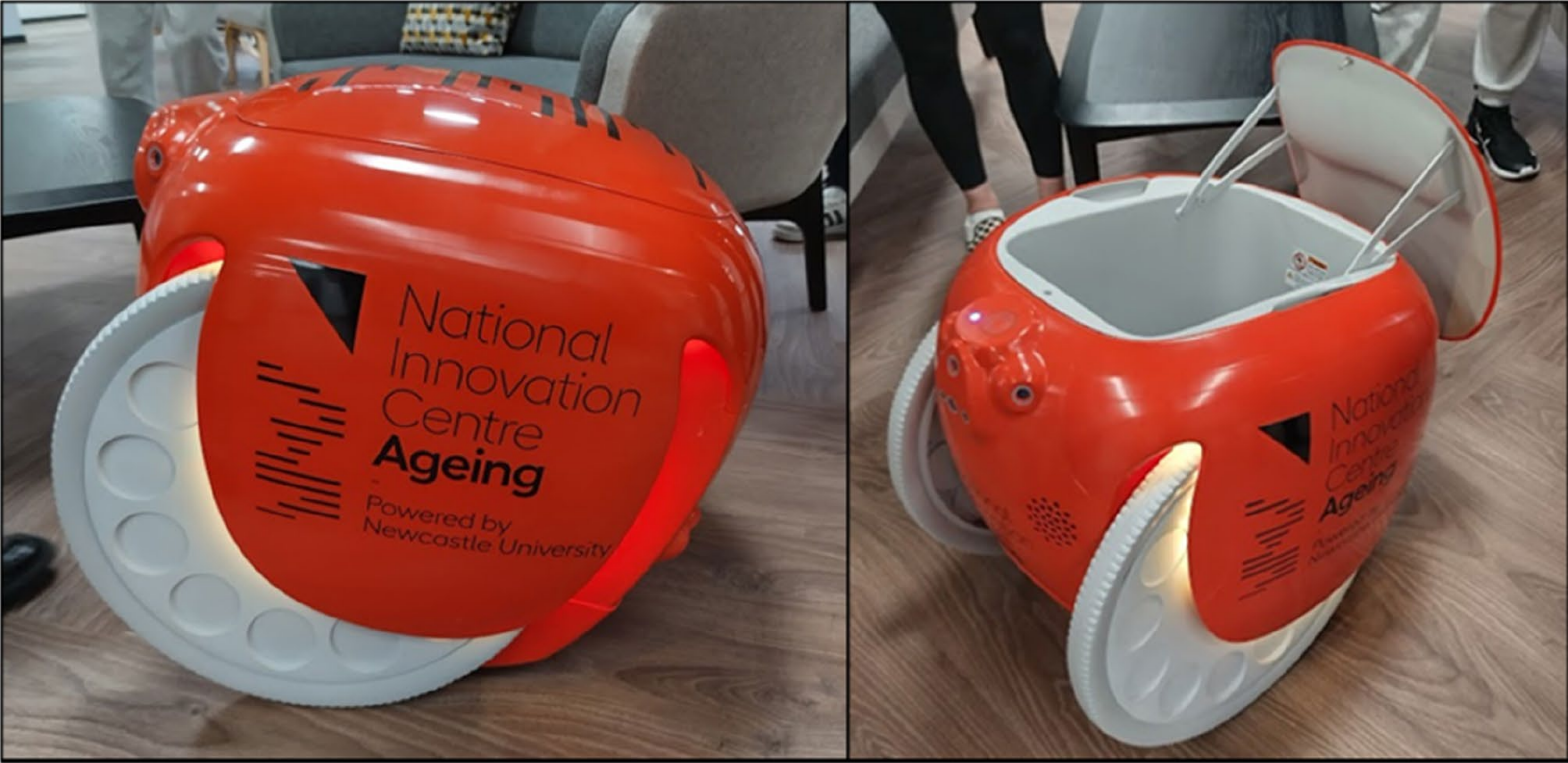
The human-following robot (Gita) used in this study.

The human-following robot adopted in this study is the Piaggio Fast Forward’s Gita robot, as shown in Fig. 1. It is designed to follow humans while also being able to carry cargo. This robot can move on its own wheels and can carry up to 18 kg of items in its internal cargo compartment as it follows its user. The robot operates with depth and colour sensing technology to track and respond to obstacles in its environment while following its user. This device is suitable for use both indoors and outdoors, and it is commonly used in urban settings. The robot is capable of navigating ramps with a maximum gradient of 16%, similar to how a wheelchair or stroller would. However, it needs human aid to move through stairs and kerbs. It has a travel range of up to 4 h (12 miles) on a full battery charge, and its maximum speed is 6 mph, which it adjusts based on the user's pace.

### Participants

To be eligible to participate in this study, a participant was required to be aged 60 years and over, be healthy, and live independently or with a partner. The age threshold of 60 years has been widely adopted in defining older people^34–36^ and it has also been used by previous research about ageing and mobility technologies^6,14,37^.

**Figure 2:**
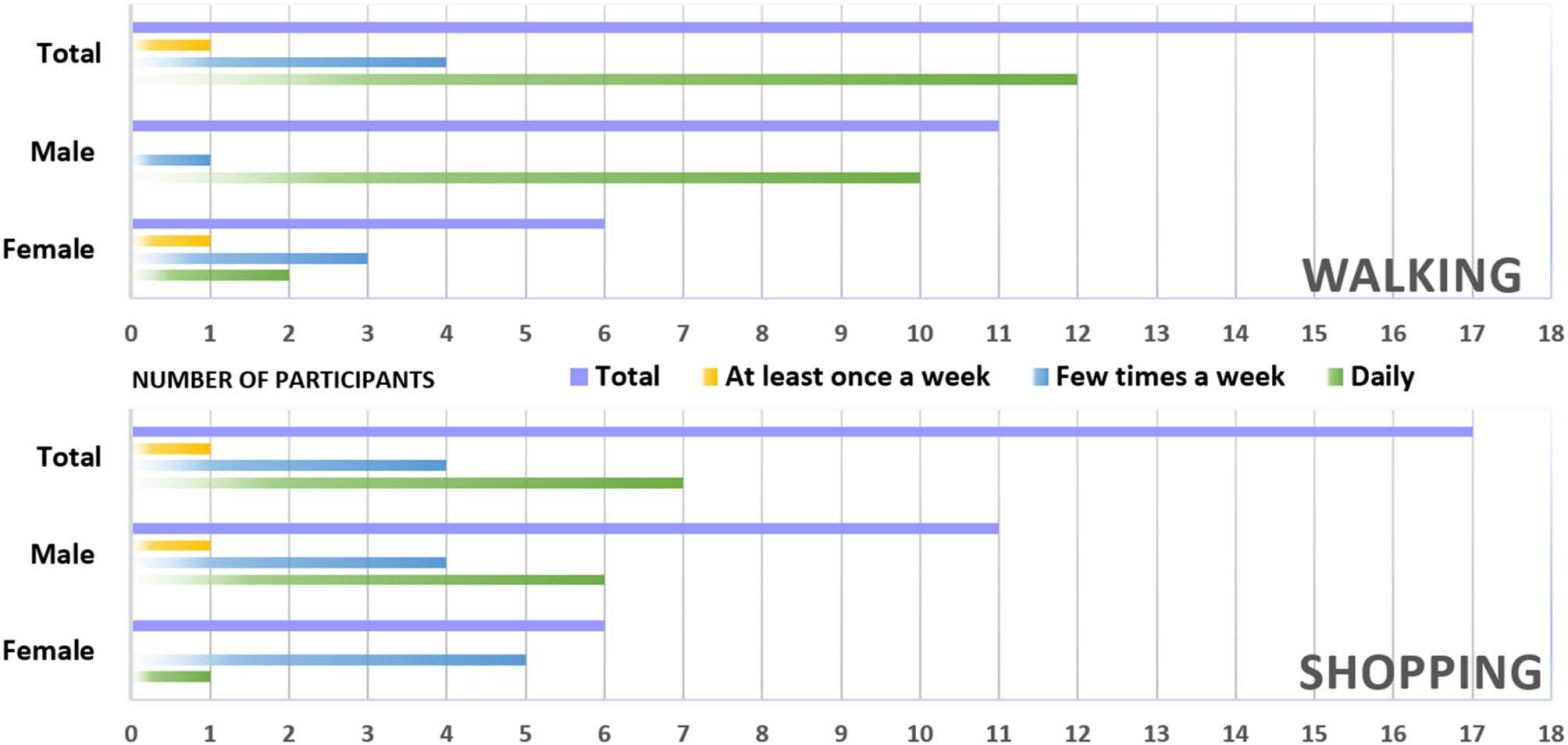
Walking and Shopping frequency of participants.

Participants were recruited across the UK and from a user group of older people called the U3A (University of the Third Age, an organisation aimed at the education and stimulation of older adults). In total, 17 older people (mean = 75.29 years, min = 61 years, max = 84 years, SD = 6.85 years; 6 male, 11 female) participated in this study. The sample size was determined based on the approximate point of data saturation, where the data collection completed once no future new information was obtained from additional interview sessions^7,38^. The participants’ walking and shopping habits is displayed in Fig. 2.

### Research design and data collection

The nature of this study is qualitative and exploratory in accordance with the research aim, which is to expand and enhance comprehension about the needs and requirements of older people regarding the human-following robot. Qualitative research is not for generalization but endeavours to provide a comprehensive and contextualized comprehension of the human experience^39^. To collect qualitative data, focus groups and individual interviews are the most common methods^40,41^. The semi-structured individual interviews were deemed to be more suitable for this study as that participants could be provided more time to fully understand the robot use cases and interview topics and articulate their thoughts^7^.

**Figure 3:**
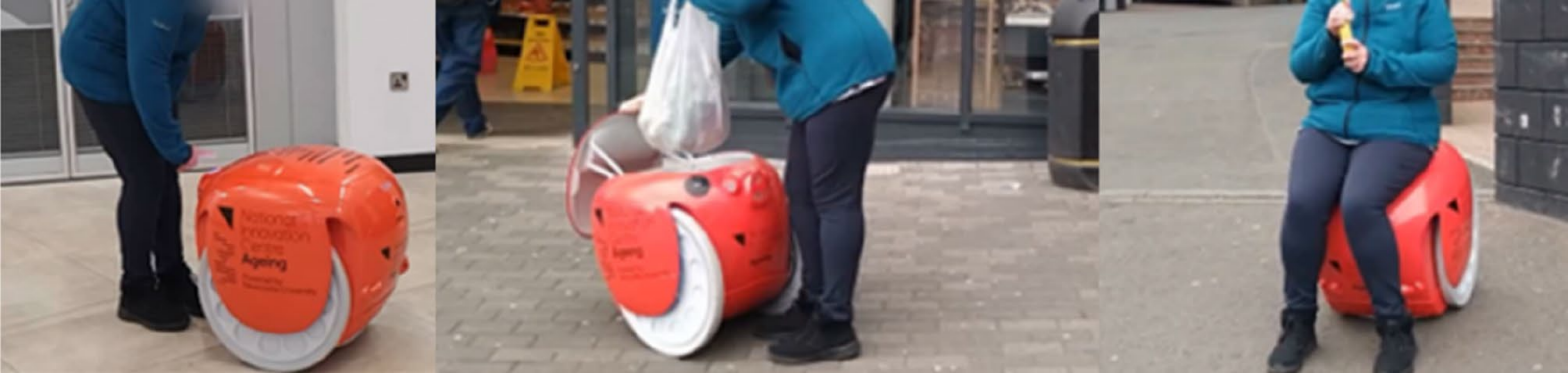
The screenshots of the video featuring a short shopping trip using the human-following robot (Gita).

Before expressing their views regarding the key topics of this study, participants would need to understand and be familiar with the human-following robot use cases in outdoor environment. A video of a short shopping trip with the robot was used, as shown in Fig. 3. At the beginning of the video, the robot is shown in a seated position through a 360° rotation. In the following scene, the user is seen approaching the robot and pressing the Ready/Park button and the Pair/Unpair button to make it stand up and pair. After that, the robot proceeds to follow the user. Subsequent scenes portray the robot accompanying the user on a walk to a local shop in an urban setting. Afterward, the user enters the shop with the robot following and proceeds to walk around while the robot follows her, moving through the shop aisles as she selects her desired items. After this, the user leaves the shop and places her shopping in the cargo hold of the robot. After pairing with the robot, she then walks towards a pedestrian crossing nearby. In the following scene, the user is seen heading towards a local football stadium before pausing to sit and rest on the robot while eating one of the bananas she bought. The final scenes show the user and robot returning to the building where the robot is stored.

Interviews would be restricted to a 30-min total duration, including completion of the demographic survey. A participant information sheet and the supplementary information sheet was provided to the participant before the interview.

### Ethical considerations and data analysis

**Figure 4:**
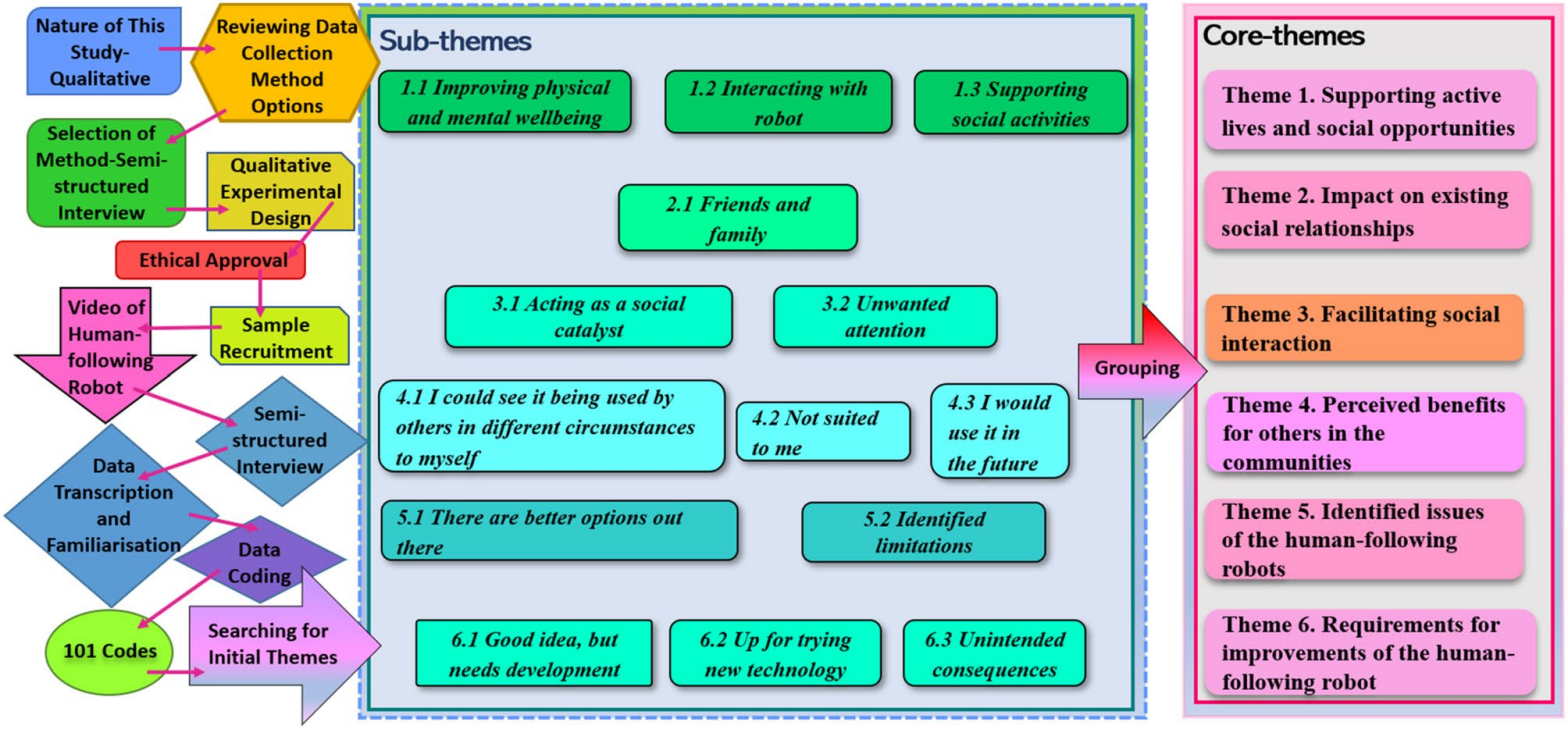
Illustration of research process and the thematic analysis.

Ethical approval was obtained prior to the data collection of the study. All the participants were informed that their participation is entirely voluntary, and they are free to withdraw from the study at any time for any reason. And access to the data only available for those involved in this study. Before data analysis, all interviews were transcribed. And all participant identities were ammonised. The collected qualitative data was analysed using the Thematic Analysis^42^. The research process as well as the Thematic Analysis is illustrated in Fig. 4.

### Informed consent

We confirm that informed consent from all subjects for publication of identifying information/images in an online open-access publication. Informed consent was obtained from all subjects. All experimental protocols were approved by the Newcastle University Ethics Committee. All methods were carried out in accordance with relevant guidelines and regulations.

## Results and discussions

The 17 older people’s interview transcripts resulted in 101 codes in total, they were grouped into 6 key themes and 14 sub-themes. Table 1 summarises the thematic analysis.

**Table 1:** Summary of thematic analysis.

Theme and subthemes	Code and frequency of codes (participants and mention during interview)
Theme 1. Supporting active lives and social opportunities	
1.1 Improving physical and mental wellbeing	a. Be able to carry on independently (n = 1 [0M, 1F], f = 1) b. Could give confidence on walking trips (n = 4 [2M, 2F], f = 6) c. It would get me out more (n = 5 [0M, 5F], f = 13) d. To keep your mobility (n = 3 [1M, 2F], f = 9) e. Use as a seat is a positive (n = 9 [3M, 4F], f = 14) f. Useful for shopping locally (n = 4 [1M, 3F], f = 6) g. Would encourage me to walk (rather than drive) (n = 2 [0M, 2F], f = 7) h. Would take the pressure off walking by carrying (n = 6 [1M, 5F], f = 15) i. Alt. Use—it would be useful for carrying stuff to support walk (n = 4 [2M, 2F], f = 4)
1.2 Interacting with robot	a. Applies anthropomorphic or zoomorphic characteristic to robot (n = 7 [3M, 4F], f = 14) b. Wouldn't take for a walk (n = 1 [0M, 1F], f = 1)
1.3 Supporting social activities	a. Alt. Use—it would be useful for Library Books (n = 3 [0M, 3F], f = 5) b. Alt. Use—other suggestions (n = 3 [2M, 1F], f = 8) c. Alt. Use—picnic (n = 4 [1M, 3F], f = 4) d. Alt. Use—sports (n = 5 [2M, 3F], f = 5) e. Alt. Use—use for travelling in airport or as a suitcase (n = 4 [0M, 4F], f = 6) f. Alt. Use—use to carry tools for recreational activities (n = 3 [1M, 2F], f = 6)
Theme 2. Impact on existing social relationships	
2.1 Friends and family	a. Alt. Use—taking something to someone (n = 4 [1M, 3F], f = 5) b. It would make no difference to how often I saw friends or family (n = 16 [6M, 10F], f = 29) c. Other people might be thinking of getting one (n = 2 [1M, 1F], f = 3) d. Others visiting the robot (n = 3 [1M, 2F], f = 6) e. Telling others about robot (n = 2 [1M, 1F], f = 3)
Theme 3. Facilitating social interaction	
3.1 Acting as a social catalyst	a. I have mixed feelings about others asking about robot (n = 5 [3M, 2F], f = 10) b. I would ask someone about it if I had seen before (n = 13 [3M, 10F], f = 18) c. I would welcome others asking about the robot (n = 7 [1M, 6F], f = 26) d. I wouldn't have asked about it before interview (n = 3 [2M, 1F], f = 4) e. I wouldn't mind others asking about robot (neutral) (n = 5 [2M, 3F], f = 15) f. Would be a talking point (n = 2 [0M, 2F], f = 4) g. Would be a way of encouraging social interaction with others for me (n = 4 [0M, 4F], f = 7) h. Would generate attention from others (n = 7 [2M, 5F], f = 9)
3.2 Unwanted attention	a. Concern—other people undesirably interacting with robot (n = 1 [1M, 0F], f = 1) b. Concern—what other people think (n = 5 [2M, 3F], f = 12) c. Concerns with security of robot and contents (n = 6 [3M, 3F], f = 25) d. It might make me vulnerable (n = 4 [1M, 3F], f = 11) e. Would get tired of explaining to others (n = 3 [2M, 1F], f = 5)
Theme 4. Perceived benefits for others in the communities	
4.1 I could see it being used by others in different circumstances to myself	a. Depends on individual living circumstances (n = 7 [2M, 5F], f = 14) b. I can see it in care homes, assisted living and hospital (rehabilitation) settings (n = 4 [2M, 2F], f = 13) c. I live too far to walk to the shop (n = 3 [0M, 3F], f = 7) d. Others—I could see it being used by other elderly people (n = 8 [3M, 5F], f = 25) e. Others—I could see it being used by people who are not elderly (n = 5 [3M, 2F], f = 9) f. Others—I could see it being used by people with physical impairments (n = 5 [1M, 4F], f = 16) g. Others—might help wheelchair user (n = 2 [0M, 2F], f = 3)
4.2 Not suited to me	a. I don't need it because I'm fit and healthy (n = 10 [4M, 6F], f = 24) b. It could actually make things more difficult for me (n = 2 [0M, 2F], f = 2)
4.3 I would use it in the future	a. If I, or others, could no longer drive (n = 6 [3M, 3F], f = 11) b. If my health degrades (n = 7 [2M, 5F], f = 12) c. Maybe I'd use in the future if my living circumstances changed (n = 6 [1M, 5F], f = 14)
Theme 5. Identified issues of the human-following robots	
5.1 There are better options out there	a. Alt. Preferred—I'd just shop online (n = 3 [1M, 2F], f = 4) b. Alt. Preferred—it doesn't match up to a mobility aid or wheelchair (n = 4 [2M, 2F], f = 6) c. Alt. Preferred—it doesn't match up to a shopping trolley (n = 7 [2M, 5F], f = 24) d. Alt. Preferred—it doesn't match up to the car (n = 3 [2M, 1F], f = 4) e. Alt. Preferred—other (n = 2 [1M, 1F], f = 2) f. Technology a step too far (n = 4 [1M, 3F], f = 6)
5.2 Identified limitations	a. But it doesn't help with walking (n = 2 [1M, 1F], f = 6) c. Concern—capacity and size of hold (n = 8 [4M, 4F], f = 16) d. Concern—cost (n = 6 [3M, 3F], f = 18) e. Concern—getting into car or onto public transport (n = 7 [1M, 6F], f = 17) f. Concern—I'd have to plan my route (n = 9 [3 M, 6F], f = 18) g. Concern—Kerbs and steps (n = 17 [6M, 11F], f = 49) h. Concern—'looking after' robot (n = 2 [1M, 1F], f = 6) i. Concern—manoeuvrability (n = 5 [1M, 4F], f = 9) g. Concern—technology and it's limitations (n = 12 [4 M, 8F], f = 24) h. Concern—using in busy or crowded area (n = 7 [2 M, 5F], f = 15) i. Concern—weight of robot and lifting it (n = 12 [4M, 8F], f = 31) j. Concern—where to keep it (n = 8 [2M, 6F], f = 12) k. Concern with terrain limitations (n = 10 [4M, 6F], f = 29) l. Concerns—other practical (n = 2 [1M, 1F], f = 6) m. I am sceptical and don't think that I would use it (n = 9 [4M, 5F], f = 21) n. I'm worried about practicalities (n = 7 [4M, 3F], f = 12) o. Limited use and benefit (n = 7 [3M, 4F], f = 13) p. More negatives than pluses (n = 6 [3M, 3F], f = 10)
Theme 6. Requirements for improvements of the human-following robot	
6.1 Good idea, but needs development	a. Additional and changed features—other suggestions to improve for elderly (n = 7 [3M, 4F], f = 15) b. Additional feature—alarm system (n = 4 [2M, 2F], f = 6) c. Additional feature—handle and manual manoeuvrability (n = 6 [1M, 5F], f = 18) e. Additional feature to warn others of robot (n = 4 [1M, 3F], f = 8) f. Additional features for added uses and benefit (n = 3 [2M, 1F], f = 4) g. Alt. Use—more for retail high street or shopping centre shopping (n = 3 [0M, 3F], f = 13) h. Change to handle kerbs and steps (n = 7 [3M, 4F], f = 19) i. Change to handle more types of terrain (n = 4 [1M, 3F], f = 8) m. Gita has advantages over alternatives (n = 5 [1M, 4F], f = 6) n. I see the potential benefits (n = 13 [5M, 8F], f = 51) o. I would consider using Gita (n = 1 [0M, 1F], f = 1) p. It's a good idea but needs improvements to be made fit for purpose (n = 5 [2M, 3F], f = 6) q. Personalisation of features and colour (n = 5 [2M, 3F], f = 8) r. Shop mobility type loan (n = 2 [0M, 2F], f = 5) s. Using within supermarket (n = 5 [3M, 2F], f = 6)
6.2 Up for trying new technology	a. A trial period with Gita would be useful (n = 2 [1M, 1F], f = 3) b. Experience with other robots and technologies (n = 6 [3M, 3F], f = 11) c. I think it looks fun (n = 4 [2M, 2F], f = 11) d. Interest in and acceptance of technology (n = 6 [5M, 1F], f = 14) e. It's a novelty at the moment (n = 9 [2M, 7F], f = 12) f. You don't know until you've tried it (n = 2 [1M, 1F], f = 7)
6.3 Unintended consequences	a. Concern—hazard for vehicles when crossing road (n = 3 [1M, 2F], f = 8) b. Concern—hazard to others (n = 6 [2M, 4F], f = 11) c. Concern—insurance implications (n = 2 [1M, 1F], f = 2) d. Concern—comfort of seat (n = 1 [0M, 1F], f = 2) e. Concern—operating in different light and weather conditions (n = 3 [1M, 2F], f = 11) f. Concern—range, battery life and charging (n = 6 [2M, 4F], f = 15) g. I would have to change my shopping trips (n = 7 [2M, 5F], f = 13) h. I wouldn't want to rely on it (n = 2 [0M, 2F], f = 3)

*n* number of participants, *M* male, *F* female, *f* number of code mentions.

### Theme 1. Supporting active lives and social opportunities

The first theme is that a human-following robot could support active ageing, through encouraging older people to get out of the home and walk more and through facilitating older people to participate in social activities. It consists of 17 codes within three sub-themes. Example quotes were outlined in Table 2.

**Table 2:** Example quotes of Theme 1 Supporting active lives and social opportunities.

Theme 1. Supporting active lives and social opportunities	
Sub themes	Example quotes
1.1 Improving physical and mental wellbeing	*“I think it would be good for shopping. For going down and walking back. To get some daily exercise. To go to shop. Or to the post office or to the Boots, the chemistry, and bring back what you need without having to carry it. To me, that's the advantage of it, I think.”—****F7, Aged 74*** *“Like today I could have done with this robot because I went into town. It's about a 15, 20-min walk. Bought lots of groceries and had to walk back, and I felt very old coming back.”—****F5, Aged 82*** *“…it doesn't matter how big or small the items are. See XXX's mum, she had a walking stick. So, when she had her shopping in the other hand, she felt vulnerable. Because if she fell, she had nothing to catch herself. Whereas that would have released that. […] I mean, somebody might say “Well, if they've got a Zimmer frame with a seat, you've got the same sort of thing”, but you haven't because you can't put your… you could put your shopping on the seat, but then you couldn't sit on it. So that’s both.”—****F7, Aged 70*** *“But if you wanted to walk, somewhere every day but couldn't because maybe the weather was a problem. You needed to carry some coats or brollies, or whatever. Maybe that would be of use.”—****M4, Aged 82*** *[M1]: “[Referring to friend] was taken out from Christmas lunch by the grandchildren, slipped on snow, hit her head and she lost the sight of one eye. And it’s ruined her self-confidence. I think, unnecessarily really. She’s more capable than she thinks, but she’s scared. I think something like this could be a lifeline for her. […]* *[Interviewer] “Yeah, so to just give some added confidence really?”* *“Yes. This is yours, it works for you.—****M1, Aged 75*** *“I do think it might encourage me… it would definitely encourage me to walk more than take the car […] I mean if I had that sitting in my… in my hall or whatever […] then I’d probably think “Yeah, I’ll just take it and go and do my shopping.””—****F1, Aged 81*** *“For me? If I had one? I might go out more often. And if you were holding on to that, you would probably feel more secure because it’s not wobbling. It is sturdy. You might feel a little steadier with that.”—****F5, Aged 82***
1.2 Interacting with robot	*[In reference to user following] “Like a dog. Or a cat! […] Well, you know, you’ve seen the dog carrying the newspapers, this is the new version!”—****M1, Aged 75*** *“…isn't it clever?!”—M3, Aged 84* *“I don't see that it would be something that I would take for a walk.”—****F1, Aged 81***
1.3 Supporting social activities	*“But also, if you are going for a picnic somewhere. You know, taking a grandchild. You could put your picnic stuff in there, without having to carry it because a picnic can be heavy.”—****F7, Aged 70*** *“Oh, I know what it could do! It could hold the books. I run a book club. It could hold books. But, can I get to the library, without going up and down?”—****F8, Aged 72*** *“Actually, it did cross my mind when I was watching the video there, that wondered how a coach company would feel about putting it in their luggage rack. In their luggage thing down the side. So that I can actually go away for a weekend and put all of my clothes in it.”—****F11, Aged 69***

Many participants presented views that the robot would physically support them whilst on a walk, citing that it would alleviate pressure whilst walking through removing the need to carry (n = 6, 1 male, 5 female) and offer as a seat for respite when needed by an elderly user (n = 9, 3 male, 6 female), especially for local shopping trips (n = 4, 1 male, 3 female). Several participants also suggested that the robot would be useful for carrying other items to support them whilst on their walk (n = 4, 2 male, 2 female), such as a drink or coat. With this, some participants expressed that having a human-following robot could improve confidence whilst walking (n = 4, 2 male, 2 female) and help older people to maintain their mobility and independence as they aged (n = 3, 1 male, 2 female). These findings suggest that the robot would be multi-functional for older people, this could be used to demonstrate the commercial viability of human-following robots for the older generation, as per the suggestions of Bedaf, Gelderblom and de Witte^43^. Several participants stated that the robot could encourage them to go out more often (n = 5, 5 female) and some indicated that having a human-following robot would encourage them to walk rather than drive for a trip (n = 2, 2 female). This corresponds to previous findings that robot itself could be promote increased intentions to walk, as suggested by Karunarathne et al.^22^. These findings also indicate that human-following robots could support the active ageing, both by inspiring exercise for the maintenance of physical and mental health^44^ and by encouraging increased ventures outside the home.

Participants also suggested alternative uses related to carrying objects for social and recreational activities, including sports equipment such as for golf and bowls (n = 5, 2 male, 3 female), picnics (n = 4, 1 male, 3 female), and library books (n = 3, 3 female). Other suggestions related to transporting gardening tools and art supplies (n = 3, 1 male, 2 female). These findings emphasize that the human-following robot could support the social participation of an independently living older adult, thus facilitating active ageing. Likewise, this finding reiterates that the robot could be multi-functional for elderly citizens, bettering the commercial viability of human-following robots for the older people^43^. Additionally, a few participants posed that a human-following robot could aid them on long-distance trips and support holiday activities (n = 4, 4 female), either as a ‘robotic suitcase’ or within the airport, which indicates that future research could further explore the use case of the human-following robotic suitcase among older people. In addition, this study has found evidence to suggest that the robot may be perceived socially by older people. Older people were seen to apply anthropomorphic and zoomorphic characteristics to the robot, such as by employing gendered terms. This finding suggests that the non-anthropomorphic robot may be perceived as a socially^45^.

### Theme 2. Impact on existing social relationships

The second theme focusses on whether a human-following robot would offer any support for the social relations between an independently living older person and their friends and family. The sub-themes and example quotes are summarised in Table 3.

**Table 3:** Impact on existing social relationships.

Theme 2. Impact on existing social relationships	
Sub themes	Example quotes
2.1 Friends and family	*“I can't think that it would make much difference to friends and family coming to see us. I can’t think that. But maybe for me going out to see them if I needed to take something and they were local. It might be good for that.”—****F7, Aged 74*** *“No. Why would it? I mean, I might say “Would you like to come and see my robot?”, but that would only be the once! [Laughing] […] I mean, that’s very sad, isn’t it?!”—****F3, Aged 76*** *“I don't think so. No. I don't see them coming around to visit the robot. If you had a dog, it'd be different. […] don’t think it has the same drawing power. It probably would when you're out, you know, in terms of people being interested to know what it was—“What on earth”, that sort of thing. But I don't see anyone coming to visit you because you've got it. Unless someone else is thinking of getting one.”—****M3, Aged 84*** *“I mean, I have been talking about it. I have a son in the States who is… Who is a computing architect, and a brother… He's a design engineer, and I sent them both pictures, I mean, and they were both interested.”—****F1, Aged 81***

In general, participants did not feel owning a robot would make a difference to how often they saw friends or family and would have no significant impact on their existing social relationships. Several participants went on to express that the only differences they could foresee was if they were taking something to someone (n = 4, 1 male, 3 female) or if someone was considering one for themselves. A couple of participants commented on others ‘visiting the robot’ but anticipated no lasting effect. As such, it is suggested that any incidence of this is attributable to the novelty effect^46,47^ and would have no lasting impact on interactions with family and friends. The only long-term difference noted by older people in the study was if the robot was to facilitate them taking something to someone they knew, though it is considered that this would be the use of the robot for convenience rather than the robot encouraging the social engagement. This finding is different from previous findings suggesting that robots can encourage increased familial social interaction. This could because the robots adopted in the previous studies are designed specifically with the purpose to interact with humans^48,49^.

### Theme 3. Facilitating social interaction

The theme 3 consists of 15 codes and two sub-themes, ‘Acting as a Social Catalyst’ and ‘Unwanted Attention’, this theme shows the advantages and disadvantages of the robot attracting attention from other members of the community in a public environment, as perceived by older people. The sub-themes and example quotes are summarised in Table 4.

**Table 4:** Example quotes of Theme 3. Facilitating social interaction.

Theme 3. Facilitating social interaction	
Sub themes	Example quotes
3.1 Acting as a social catalyst	*[F6] “I’d think you’d get a lot of people looking at you.”* *[Interviewer] “Yes, you do.”* *[F6] “Now, for myself, that wouldn't bother me! [Chuckles] And I think would be a great talking point!”—****F6, Aged 70*** *“I enjoy talking to people, and I think that people should talk more, quite honestly. And something that invites curiosity and I was out with it, then I would think “This is a novelty, of course I’m prepared to talk about it”, just as my friend at the moment who has got an adult tricycle. That invites comment…* *…I think it is important for people to, sort of, to engage with other people around them. Talk with them. So, you know, if I had something like that and was out, and therefore I was talking to people and they were talking to me, I would feel that a bonus on the social side. For them, as well as me.”—****F9, Aged 81*** *“And I could see it as being a sort of a way… I think, a way of making friends in the sense that I am sure my neighbours would want to chat about it.”—****F1, Aged 81*** *“It wouldn't bother me, but I wouldn't be going out to get a robot just to draw attention to myself. If I wanted to do that I could… I could paint myself into a robot and pretend to be a robot myself. It’s not, you know, it's not like I would want to go out to meet and talk to different people just because I’ve got a robot.”—****M6, Aged 61*** *“Mixed feelings. Mixed. You know, some will be genuinely think it's a bit of fun, but you will get you as a course who’ll think ‘What a load of rubbish’, you know. “What are you doing with that walking behind”, this sort of thing.”—****M5, Aged 79*** *“I would have stayed well clear of them. [Laughs] To give the robot some space. I mean in terms of the sensors and things like that, I don't want it to start following me by accident.”—****M6, Aged 61***
3.2 Unwanted attention	*“My wife is a bit self-conscious, and I think she’d rather be dead than seen with one of those travelling with her.”—****M2, Aged 83*** *“But there again, you are announcing that I’m an older person and…. I think people will think that it's not for shopping, they’ll think that you're being guarded.”—****F5, Aged 82*** *“Well, I wouldn't really mind if people came up to chat and say hello, but if it's people who are up to no good…”—****F5, Aged 82*** *“…What would happen if somebody decided that they wanted they wanted to take it with them? You know, if somebody thought “Gosh, that’s a useful thing. I would like that, for myself”. Yeah, I mean, I wouldn't want to try and stop anybody taking it. That would be an issue really.”—****F1, Aged 81*** *[F9] “Well, I don’t think I’d take it out when I’m out at night* *[Interviewer] “No?”* *“No. I think, you know, there are some people out at night and, you know, no regard for me whatsoever, and you know just like that. There might be some out who “Oh, oh. She's got one of those things! We’ll go out one night and…”.”—****F9, Aged 81*** *“I think I’d be more bothered by people trying to interact with it themselves. I think that could be a problem if you if you come, you know, a big gang of teenage boys or something that they're mucking around, and you know they would want to play with it or have a sit down on it while it's moving […] I can see that being an issue…”—****M6, Aged 61***

Many participants showed positive attitudes towards the potential attention gained through the use of the human-following robot (n = 7, 1 male, 6 female). Some participants considered that the robot would act as a talking point for conversations with others in their community, perhaps even fostering new social relationships. Other participants provided a more impartial stance towards interactions with members of the public, citing that they would not mind others asking or that it would not bother them (n = 5, 2 male, 3 female). These findings are in accordance with Hudson et al.^49^, who found that older people formed new friendships through taking their robotic pet to public spaces subsequent to the social interactions prompted. This finding is also comparable to those presented by studies on dog walking^50,51^. Therefore, this implies that a human-following robot could stimulate new human social relationships for an elderly user with others in the community and support active and healthy ageing^15^. However, further research is needed to validate the potential of the human-following robot on facilitating new social relationships for older people using naturalistic research methods.

Some participants had reservations about the prospect of the robot gaining the attention of others if used in a public environment. Particularly, a few shared that they would be concerned about how they would be perceived by others when using the robot (n = 5, 2 male, 3 female). With this, several participants voiced that the robot could make them feel more vulnerable (n = 4, 1 male, 3 female) and a further participant disclosed that he was concerned other people would interact with the robot undesirably (n = 1, male). A few participants also shared that they would become tired of members of the public asking about the robot after a while (n = 3, 2 males, 1 female). Therefore, it is important for further research to explore any potential measures to address these concerns, for example, future iterations of the robot could implement a security system which could allow the end-users to reach out to a remote operator if needed^52^. Further, the results suggest that some older people would feel self-conscious using the robot in a public environment. It is important for future research to adopt quantitative methodology to further examine the impact of feeling of self-consciousness on the trust, acceptance, and intention to use the human-following robot among older people.

### Theme 4. Perceived benefits for others in the communities

The Fourth theme consists of 12 codes and 3 sub-themes. Older people of this study could see the potential benefits of the human-following robot for others in the community, or themselves if their circumstances were to change in the future. The sub-themes and example quotes are summarised in Table 5.

**Table 5:** Example quotes of Theme 4. Facilitating social interaction.

Theme 4. Perceived benefits for others in the communities	
Sub themes	Example quotes
4.1 I could see it being used by others in different circumstances to myself	*“I could see it maybe being useful for somebody that's… See I can still carry bags, you know. Because I can still sort of carry stuff so… Yeah, if somebody is a bit… their mobility has gone a little bit and move on a stick, then you've got one less hand, haven’t you? But at the moment, touch wood, you know my health’s not too bad, but if your health declined, then it could be useful.”—****F4, Aged 62*** *“Oh, I think is it's really good for the older generation. Let’s face it, we're living longer. But, and we have this talk quite frequently in places like the WI, as you get older… I mean, I’m one of the lucky ones that I can still get around, I can still do everything. But, as you become more limited, you're looking for things that are going to help.”—****F6, Aged 70*** *“I’ve got a friend with terminal cancer, who is quite weak. She goes into remission but, even when she's in remission she's quite weak. Something like that would be brilliant. I’ve got other friends who have got Parkinson’s who would find this ideal.”—****F6, Aged 70*** *“Once you've got hold of a guide dog, and if you have to have a stick as well, then your hands are full and I can imagine that it’d be very good for people who are blind. Or people who are unable to… You know, have that cognitive reaction, you know with that with them. Yeah, I think it could be very useful […] For people who are indoors a lot and are frightened to go out because they can't manage a stick and a bag.”—****F2, Aged 72*** *“I’m just thinking though, it might be useful in a hospital setting where, you know, you have, you know, people that are not fully mobile and, you know, that they want to do some exercise around the hospital and they can just follow them around and make sure that, you know, they’ve got their own possessions, or the medication that they need, you know behind them, and they can take a rest whenever they need to* *Something like that I could see. You know, that might be a really useful thing, application of it. But in a setting where that's going to be much more acceptable and where you're not going to get any stairs or steps or anything like that.”—****M6, Aged 61*** *[F11] “I think if it's in an area where there was a large elderly complex of, not just bedrooms, but their own sort of front door* *[Interviewer] “Yeah, like the kind of assisted living?”* *[F11] “Yes. Then having one or two for people to use, I think would be quite useful. […Suppose] you wanted to go out for a shop, you could actually say “Right , I’m gonna pick this one […] I can pick this and go to do my little shop or walk or whatever. And I’ve got a seat there if need be. If I get tired, I can sit and rest. I can carry a bottle of juice or water or whatever in it.” You know, that sort of thing. And then come back again, so I do think that there would be a use for them in that sort of situation.”—****F11, Aged 69***
4.2 Not suited to me	*“I’m quite sceptical as to the benefits that I would gain from use of the Gita. I am very fortunate, in that, for my age, I am comparatively active and able to get a good amount of exercise.”—****M5, Aged 79*** *"I wouldn't have any use for it at all. Fairly straightforward, because at the moment, touch wood, I haven’t got any problems. But you never know what lies in the future, do you?!”—****M4, Aged 82***
4.3 I would use it in the future	*“I think… If I wasn’t allowed to drive […] then yes. Something like this would be… well what’s the alternative? Plod along the road carrying two bags. And something like this, that does the bag carrying, yes please.”—****M1, Aged 75*** *“…it would be when I had a problem carrying weights. Or I’d lost my driving licence. Or could no longer afford a car, you know. Couldn't see properly to drive, anything like that. Any reason I couldn’t drive. I’d probably be interested in that it could take the load off me.”—****F11, Aged 69*** *“I expect that, as I get a bit older, I will want to live somewhere which is much nearer to shops, and then it would become more useful because I would probably be less able.”—****F2, Aged 72***

This study found that many older people who live independently do not feel they need a human-following robot at this stage, as they perceive themselves to be in good health and relatively mobile, with no issues carrying items. Some participants that felt they did not need the robot at present, but many revealed that it could be more useful if their circumstances changed in the future (n = 12, 3 male, 9 female). Additionally, a few of the participants who specified that the robot would not be of use to them now, for instance given that they lived in a rural environment, supposed that the robot could helpful if their living situation changed, perhaps by relocating. Typically, this was reasoned with if their health declined to the extent that they would need additional support (n = 7, 2 male, 5 female) and coupled with driving cessation (n = 6, 3 male, 3 female). This finding could be interpreted as that the apparent usefulness of a robotic technology is a key determinant in whether an older person will accept a robot^53^. This builds on existing evidence of the heterogeneity of the elderly population^31,32^, agreeing with the argument that older people have a diverse range of needs in relation to the support required from assistive technologies and robots^6,54^. This creates a need for future research to identify the potential socio-demographic factors that affect older people’s acceptance, trust and adoption of the human-following robots.

Similarly, many who felt that the robot was not suited to their situation indicated that they could see it being used by others in distinct circumstances from their own, with most participants suggesting other user-types that the human-following robot could support (n = 15, 6 male, 9 female). Typically, these were other elderly people with acuter mobility needs (n = 8, 3 male, 5 female) and those with physical impairments or disease (n = 5, 4 female, 1 male), with participants repeatedly offering friends and relatives who they thought the robot could benefit. A few participants also suggested uses for the robot by non-elderly people (n = 5, 3 male, 2 female). With the robot offering physical support, these suggestions were expected given the inextricable links between physical health, mobility and wellbeing^55^, and their importance in relation to independence and quality of life. This finding indicates that there is a case for the usage of human-following robots within parts of the independently living older population but reiterates that the design of robot should consider the needs and requirements of the different user groups.

Moreover, some participants presented ideas for using the robot in settings different to the use-case presented in the interview (n = 4, 2 male, 2 female), including care homes, assisted living facilities, hospitals and rehabilitation centres. This finding was anticipated given the increased support required by older individuals in such settings and is consistent with robotic applications investigated in prior literature^56–58^. The findings indicated that the robot has the potential in addressing the care worker shortage presented by the ageing population^59^. These findings highlight the need for further research to explore the potential of utilizing human-following robots in hospital settings. And it would be important to evaluate the robot from the care worker perspectives.

### Theme 5. Identified issues of the human-following robots

Theme 5 comprises of 25 codes and two sub-themes. The sub-theme ‘Identified limitations’ shows the views of participants that felt that the present design of the robot has some room for improvement to meet all their needs and expectations, with participants expressed some concern over the practical limitations currently associated with the robot. The sub-themes and example quotes are summarised in Table 6.

**Table 6:** Example quotes Identified issues of the human-following robots.

Theme 5. Identified issues of the human-following robots	
Sub themes	Example quotes
5.1 There are better options out there	*“Right, so you’re looking for dropped kerbs all the time then? Which could be a problem, because … dropped kerbs are terrible. You just don't have them, you know.”—****F11, Aged 69*** *“You know how, when you have to cross roads when… when they've got the lowered pavement and things, you have to walk quite a long way to get to them. So, that would infuriate me. And I wouldn't be able to take shortcuts through alleys. And I wouldn't be able to go across the cobbles.”—****F8, Aged 72*** *“I’m not sure if it's 40lbs, it wouldn't be say ‘manhandle-able’ or ‘woman handle-able to get it from… from… As I say, if it got to a kerb, how to get up? It's alright if you've got one with a ramp down and a ramp up, but lot of places haven’t.”—****M3, Aged 84*** *“So, if you, you have to go to the shop in a car, how would you get it back into your car?[…] for my situation, it wouldn't really be much use to me if I'm not able to put it in my vehicle.”—****F2, Aged 72*** *“I’m just personally wondering about the practicalities… First thing I thought ‘Oh that’ll be great, stick the shopping in’, but then when I say if you're a crowded pavement and you’ve got to cross a very busy road. There are kerbs. You've got lift it up and down on the kerbs. I’m looking at the downside, as well as the upsides, you know…”—****M5, Aged 79*** *“It is expensive. Well, it, it was… The site I was on just gave it in dollars, but three and a half thousand dollars. Which doesn’t equate with just £100 in this country. The shopping trolleys I was looking at, some were less than £20 and certainly didn’t go over £100.”—****F9, Aged 81***
5.2 Identified limitations	*“Well, a shopping trolley is more… What’s the word?… You can put it in the car. You can leave it standing by the checkout and nobody will be interested in a shopping trolley. You can hang it on the Tesco’s shopping trolley, I mean. There’s a thing you can hang your shopping trolley on.”—****F3, Aged 76*** *“I really can’t see it being more useful than a shopping trolley for anyone I know…. I’m not trying to be miserable. I really just don’t see it as an advantage over a normal shopping trolley.”—****M2, Aged 83***

Markedly, the inability of the robot to traverse kerbs or steps was a concern for all participants, with over half of participants feeling that they would need to plan and alter their route to the shops to accommodate the robot. Linking to this, most participants expressed anxieties over the weight of the robot and felt that they would have struggled to lift the robot up a kerb or step if required (n = 12, 4 male, 8 female). This concern was also associated to lifting the robot into the car or onto public transport (n = 7, 1 male, 6 female). Similarly, the limited types of terrain that the robot can negotiate was perceived as an issue by participants (n = 10, 4 male, 6 female), particularly inability to handle grass and cobbles. The majority of participants compared the robot to alternatives (n = 13, 6 male, 7 female), either existing physical supports for aiding a shopping trip or other perceived options. Participants weighed the robot against a conventional shopping trolley bag most frequently (n = 7, 5 female, 2 male), with cost, size and manoeuvrability of the human-following robot often expressed as falling short of that presented by a shopping trolley bag. Participants also compared the robot against other mobility aids, such as wheelchairs and Zimmer frames. Further, options that would eliminate the need for an older person to walk and carry to the shop were also referred to by participants, including the car (n = 3, 2 male, 1 female) and online shopping (n = 3, 1 male, 2 female).

These findings could be possibly explained as that the robot has not been specifically designed for older people, and the potential use-case comes as an afterthought to technological development^60^. Since the current study did not involve testing older people interacting with the robot in real-world scenarios, it is crucial for future research to quantify the impact of technical limitations on older people's acceptance, trust, and comfort in using human-following robots and explore how these technical limitations could be addressed in the future iterations of the robot.

### Theme 6. Requirements for improvements of the human-following robot

The sixth theme (Table 7) captures 28 codes and three sub-themes. Most participants expressed that they could broadly see potential benefits of the robot for older people (n = 13, 5 male, 8 female), typically praising the cargo carrying functionality and a few comparing it positively to alternative options (n = 5, 1 male, 4 female). However, many participants also specified that the robot needed improvements and added features, and make it suitable to use (n = 5, 2 male 3 female) or to increase the benefit an older user would obtain from the robot (n = 7, 3 male, 4 female). The most frequently cited changes were to evolve the robot to handle kerbs and steps (n = 7, 3 male, 4 female) and further types of terrain (n = 4, 1 male, 3 female), and the addition of a handle to allow for manual manoeuvrability (n = 6, 1 male, 5 female). Other additional robot features suggested by participants were an alarm system for personal and robot security (n = 4, 2 male, 2 female), an audio or visual system to warn others of the robot (n = 4, 1 male, 3 female), a ‘lead’ type attachment to the user, a refrigerated hold, a light, a fingerprint or facial recognition security system, and an identification card with the users contact details on for in case of emergency. Several participants also expressed a desire for a personalised robot (n = 5, 2 male, 3 female), particular of the colour. Additionally, a couple of participants offered suggestions associated to implementation beyond the robot design, including a ‘shop mobility’ type service where older people could pick up a robot from a suitable location whilst shopping as needed rather than owning one individually (n = 2, 2 female).

**Table 7:** Requirements for improvements of the human-following robot.

Theme 6. Requirements for improvements of the human-following robot	
Sub themes	Example quotes
6.1 Good idea, but needs development	*“I think it's a basically a good idea. But I think it needs a bit of planning…”—****F11, Aged 69*** *“I can see how that would be very useful. When I used to walk down to the shop, the Co-op is about half a mile away and it’s all downhill, walk down to the Co-op and then be able to bring that shopping back. That would save me carrying it.”—****F7, Aged 74*** *“My main thing is that, if it was more versatile. If it was dual purpose, you know multipurpose. Say yes, automatic, it can follow me again, it could do this, but it can also be pulled along, push along trolley.”—****M5, Aged 79*** *“I think it might be useful to have some sort of alarm system on it, because if you know if you're going out, and you know you want that extra protection, maybe, maybe there's some way of activating it into alarm. So, you know, “here I am. I’m in trouble, please help. Come and help”. Something like that might be useful so it gives you added confidence in your security.”—****M6, Aged 61*** *“Maybe a little light? Yes, if it had a little light that, you know not so much for during the day, but if you're coming back in winter and it's four o'clock. It's dark. And so, you're trying to get your shopping before people come home from work so… Maybe a little light.”—****F7, Aged 70*** *“It might be useful around the town. If you went to Aberdeen shopping […] then if you could pick one up in the town somewhere and take it with you around the town it would mean you wouldn’t have to carry your carrier bags, and things like that, with you.”—****F2, Aged 70***
6.2 Up for trying new technology	*“I do love the technology, it’s a bit of fun. It is fun, yeah.”—****M5, Aged 79*** *“I don't think I would have any problems with, you know, the technology, as it were […] I mean I’m not at all worried about trying new technology and then thinking ‘well, that didn’t work very well, did it’. And I think some of my U3A people, they are very nervous about trying new technology, so I think it would have to have a trial run to see if it worked.”—****M1, Aged 75*** *“I don't think I would have any problems with, you know, the technology, as it were. No problems at all, because you know I use a motorised… battery driven golf cart, which is a very similar sort of idea. And no problems at all with it.”—****M4, Aged 82***
6.3 Unintended consequences	*“..you need it to be bright for people who are walking along because that's the other issue, I would think. How many people wouldn’t, you know, see it because they're on their phones. The number of people now, if you're walking down Northumberland Street who just do not see you. They’d be tripping over the top of it!”—****F6, Aged 70*** *“…if you're crossing roads and it gets run over or causes an accident, but I suppose…. Because it’s back just a couple of meters. There's the safety part of it, but […] the cars might not see. The cars are looking for pedestrians and cyclists, or whatever, aren't they? They're not necessarily looking for that…”****– F4, Aged 63*** *“I’d probably be interested in that it could take the load off me. But I think I would have to choose my shopping times so that it would be quieter.”—****F11, Aged 69*** *“If it's got four hours of charge and 12 miles possibly you wouldn't have to be in the position of it running out, unless you’ve forgotten to charge it! With that, well….! Because elderly people do tend to forget things.”—****F3, Aged**** 76*

A number of participants showed interest towards the robot and its technology (n = 6, 5 male, 1 female), with a few citing the robot as a ‘fun’ idea (n = 4, 2 male, 2 female), thus linking to the above sub-theme. Moreover, many participants commented that the human-following robot was a ‘novelty’ at present (n = 9, 7 female, 9 male). With this, a couple of participants suggested that a trial period would be useful (n = 2, 1 male, 1 female), given that older people would not know if the robot would work for them until they had tried it. Several participants also reported that they already owned or had considered purchasing other assistance robots and a further few compared the robot to technologies they were already accustomed with (n = 6, 3 male, 3 female).

In addition, many participants identified potential problems with using the robot that they felt would need to be addressed. Most frequently, the human-following robot was considered a hazard to other pedestrians (n = 6, 2 male, 4 female) and to vehicles, if the robot was to enter the road (n = 3, 1 male, 2 female). Consequently, a couple of participants speculated that insurance for the robot might be required (n = 2, 1 male, 1 female). Further issues identified related to reliance (n = 2), battery life and charging (n = 6), use in different light and weather conditions (n = 3) and changes to shopping habits (n = 7).

## Conclusion

Promoting healthy aging is not only important for the well-being of older adults but also potentially providing significant benefits to society as a whole^6^. The rapid development of human assistance and following robot has the potential to benefit older people and facilitate their health and wellbeing. However, to date, there has been limited research on the needs and requirements of older people regarding human-following robot. It remains unclear how human-following robot could be designed age-friendly and to provide tailored support to older people. To fill the research gaps, this research aimed to investigate the perceptions, attitudes, needs and requirements of older people concerning the human-following robot, to create new knowledge and novel insights that can support the design and development of age-friendly human-following robot. The key findings are as follows:

- Older adults perceived the potential of the human following robot to support healthy ageing.
- Older adults considered the robot's ability to carry items and function as a seat to be useful for supporting their independence as they age.
- Using the human-following robot was deemed by older people to have limited impact on the frequency of their interactions with friends and family.
- Older adults believed that human-following robots hold the potential for supporting such human–human social interactions of an elderly person, through encouraging increased walking trips and acting as a social catalyst in a public environment.
- The older people have voiced worries about possible negative interactions between the robot and others, and it may be worthwhile to consider future iterations of the robot that address these concerns. For example, implementing a security system that connects to a remote operator.
- Older adults presented that they did not yet need the support of a human-following robot given that they could still carry belongings or drive, but it could be useful for maintaining their mobility and independence if their health was to decline or their circumstances change in future, particularly if they could no longer drive.
- Older adults believed that the human-following robot could benefits others, including other older adults with acuter mobility needs, those with physical impairments or disease.
- The study has also highlighted older people’s requirements regarding where the human-following robot could be improved, such as enhancing its ability to navigate kerbs, steps, and different types of terrain. Other potential enhancements include adding a handle, an alarm system to ensure personal and robot security, an audio or visual system to alert others to the robot's presence, a 'lead' attachment to help guide the user, a refrigerated compartment, a light, and a security system that uses fingerprint or facial recognition technology. It may also be useful to incorporate an identification card with the user's contact information in case of emergencies, as well as exploring the possibility of a personalized robot.
- Practical limitations and cost of the robot are barriers perceived by older people to adopt the human-following robot at present.

In conclusion, this study has demonstrated that human-following robots have the potential to facilitate healthy aging and support better quality of life for older adults. However, further improvements to the design of these robots are necessary to ensure that they are accepted and utilized by older adults, and to fully realize the benefits that they offer. The current study emphasizes the importance of considering the needs and requirements of older people in the design and development of age-friendly human following robots, ensuring their acceptance and usability. The findings of this study highlighted that robot developers and innovators should consider the development of improved capabilities for handling kerbs, steps and more diverse terrain should be pursued for the robot to be a viable option for most older people. Another implication of this study is that future research as well as robot developers could future explore the additional features and design modifications required by older people in this study, including an alarm security system and features to alert other road users about the robot’s presence. Considering that cost was identified as a potential barrier to the adoption of human-following robots by older people in this study, it is recommended for future research to assess their commercial viability. It is suggested that other business models, such as the "shop mobility" model and inclusion hospital release packages that were proposed by older people in this study, could be pursued to increase their accessibility and affordability. Given that older people in this study felt a human-following robot would be a valuable option if they cease driving, it is therefore important for future research to investigate the potential impact of using human-following robot on mitigating the negative impact of driving cessation on older people’s independence and quality of life^37^. Such research would also potentially provide evidence-based knowledge on informing the policymakers about when it would be best to introduce an older citizen to a human-following robot. Additionally, considering that there are other promising future mobility applications and technologies that could improve the quality of life for older individuals, such as vehicle automation^7,10,14,37^, future research should explore how the human-following robots could work collaboratively with connected and automated vehicles to provide older people with tailored, seamless and convenient mobility services. The findings of this study could inform policymakers, robot manufacturers, and service providers about the key factors that affect the successful implementation of the robot. These factors include insurance, policies, regulations, trial periods, and training with the robot. An additional suggestion for future research is to explore whether self-consciousness is a potential barrier to the acceptance of assistive and robotic technologies among older individuals.

Although this study makes a novel contribution to understanding the acceptance of human-following robots by older people, limitations still exist. Firstly, the present study enabled older people to be familiar with the human-following robot by watching a video. Although the video provided sufficient details to allow the participants to understand the functions and use cases of the human-following robot in outdoor settings, there could be still impression gaps between watching a video of the robot and interacting with it physically. Even if the robot makes a positive visual impression, users perceptions may change after actually using it in-person. Therefore, future research is necessary to explore the gaps in older people’s impression, requirements and perception towards the human-following robot between watching a video of the robot and using it physically. Moreover, to validate the findings of the present study and gain new insights into how the robot could support and benefit healthy aging in the long term, it is recommended that future research should investigate the human-following robot using naturalistic methodology that involves observing and monitoring independently living older individuals interacting with the robot in real-world settings over an extended period.

The current study attempts to identify older people’s needs and requirements of the human-following robot using a qualitative methodology, future research has been planned to use a quantitative methodology to explore older people’s performance and perception when interacting with a human-following robot in person. The current study focused on designing age-friendly human-following robot from the user perspective. Future research could investigate the robot from control algorithm point of view. In addition, the human-following robot adopted in this study is non-anthropomorphic, so future research should examine older people’s needs and requirements for anthropomorphic robots, for example, the provided robots had arms or faces. Furthermore, future research should investigate how other demographic factors, such as gender affect older people’s requirements towards the human-following robots^61^. Finally, population aging is acknowledged as a global issue. As all older people participated in this study were based in the UK, it is important for future research to investigate how the perspectives, needs and requirements of older individuals towards human-following robots may vary in other regions around the world.

## Data Availability

The datasets generated during and/or analysed during the current study are available from the corresponding author on reasonable request.
